# Comparative analysis of pancreatic cancer burden attributable to high BMI in adults aged 70 and older: analysis of the GBD data in the United States, Australia, and Germany (2012–2021)

**DOI:** 10.3389/fonc.2025.1672013

**Published:** 2025-11-10

**Authors:** Hanwen Yang, Simeng Lei, Yangkai Fu, Bo Zhang, Zhili Ji

**Affiliations:** Department of Hepatobiliary Surgery, Beijing Organ Transplant Center, Beijing Chaoyang Hospital, Capital Medical University, Beijing, China

**Keywords:** BMI, GBD, pancreatic cancer, mortality, analysis

## Abstract

**Background:**

Pancreatic cancer (PC) is a highly lethal malignancy for which obesity is a major risk factor. With increasing global aging, the burden of PC is increasing in the elderly population.

**Objective:**

The objective of this study was to analyze the association between high body mass index (BMI) and the burden of PC in people aged 70 years and older in the United States of America (USA), Australia, and Germany during 2012-2021.

**Methods:**

BMI-related PC mortality and disability-adjusted life years (DALYs) were extracted from the global burden of disease (GBD) 2021 database for people aged 70 years and older. Trends in the burden of disease were assessed using age-standardized rate (ASR), estimated annual percentage change (EAPC), and joinpoint regression analyses, and differences between the three countries were compared.

**Results:**

From 2012 to 2021, BMI-related PC mortality and DALYs increased in all three countries, with the steepest rise in the USA (ASR from 3.12 to 3.65), followed by Australia (ASR from 2.05 to 2.65), and a relatively stable trend in Germany (ASR from 1.81 to 2.26). The increase was most pronounced in adults aged 70 years and older, with average annual growth rates of 3.05% in Australia, 0.55% in Germany (after 2019), and 1.71% in the USA. BMI-related PC mortality patterns varied by country: males had higher rates than females in the USA, while Germany showed the opposite trend. In Australia, men aged 70–74 had higher BMI-related PC mortality than women, but women had higher rates in all other age groups. All three countries exhibited rising BMI-related PC rates with age, though peak incidence occurred at different ages.

**Conclusion:**

High BMI significantly increases PC burden in adults aged 70 years and older in the USA, Australia, and Germany. Countries should tailor obesity prevention and health management strategies to their specific contexts to address the health challenges of an aging society.

## 1 Introduction

Pancreatic cancer(PC) is a highly lethal and aggressive malignancy with a 5-year survival rate of only 10% (1). PC is pathologically classified into pancreatic ductal adenocarcinoma (PDAC), acinar cell carcinoma, neuroendocrine tumors, and small cell carcinoma (2). With the progressive aging of the population, the incidence of pancreatic cancer has been steadily increasing (3). Because early-stage PC is asymptomatic, diagnosis often occurs at an advanced stage. Despite advances in surgery and chemotherapy in recent decades, BMI-related PC mortality continues to rise (4). Therefore, effective preventive measures are urgently needed to reduce PC incidence (5). Obesity is a known risk factor for pancreatic cancer (6, 7). Saeed et al. (8) found that increased BMI in young adults may heighten the risk of PDAC, and higher BMI in men is associated with an elevated risk of early-onset PDAC. Due to high-calorie diets, obesity prevalence is notably high in Western developed countries. Current pancreatic cancer research primarily focuses on all age groups globally. We utilized data from the GBD 2021 database, which provides estimates for 371 diseases and injuries and 88 risk factors across 204 countries and territories from 1990 to 2021; our analysis specifically focused on pancreatic cancer within this database. Data sources include BMI-related PC mortality registries, surveys, hospital records, and disease registries. Primary outcomes were years of life lost (YLLs), years lived with disability (YLDs), and disability-adjusted life years (DALYs) (9, 10). Age-standardized rates, calculated using the global standard age structure, ensure comparability across countries and time periods. The GBD database has generated extensive evidence on disease burden worldwide, informing health policy development, advancing medical research, and guiding public health decisions (11). Global population aging poses a significant socioeconomic challenge. This study chose the United States, Germany, and Australia for comparison as they represent high-income, high-SDI regions in North America, Europe, and Oceania, respectively, with a significant PC burden associated with high BMI (12, 13). Australia also falls within the high-incidence “High-income Asia Pacific” region (14). We analyzed data from 2012–2021 to utilize the most recent GBD information (15). Global population aging presents a paramount socioeconomic challenge. Research shows PC incidence is lower in adults aged ≥70 years compared to younger age groups (16, 17).We focused on adults aged 70 and older from these three nations, which have among the highest GDPs in their respective regions.

## 2 Methods

The GBD 2021 study is a comprehensive, multinational, multidisciplinary health research project. It compiles the latest global health data, including metrics on disease incidence, mortality, disability, and risk factors (18). The quantification of risk factors relies on the comparative risk assessment framework developed by the GBD Risk Factor Collaborators (19). Within this framework, a high BMI in adults is defined as a BMI higher than 25 kg/m^2^ (19). Data on BMI-related PC mortality and disability-adjusted life years (DALYs) were retrieved from the Global Burden of Disease (GBD) Results Tool (https://vizhub.healthdata.org/gbd-results/). A DALY equals the sum of years of life lost (YLL) and years lived with disability (YLD); one DALY represents one year of healthy life lost (20). We extracted data from the GBD portal by selecting ‘Risk Factor’ under ‘Estimate’, then ‘High body-mass index’ under ‘Risk’, and finally ‘Pancreatic cancer’ under ‘Neoplasms’. We selected the following age groups from the database: 70–74, 75–79, 80–84, 85–89, 90–94, and 95 years and older. Finally, we selected the 2012–2021 time period for analysis. Studies using the GBD database provide extensive evidence on the global burden of disease.

We estimated the annual percentage change (EAPC) in BMI-related PC mortality and DALYs to quantify average per-year trends during the study period. We calculated the EAPC for each age group. BMI-related PC Mortality and DALYs were considered to have increased if both the EAPC and the lower bound of its 95% confidence interval (CI) were positive, and to have decreased if both the EAPC and the upper bound were negative (21). We divided the study period into intervals and fitted a joinpoint regression model (22). Age–period–cohort (APC) analysis simultaneously evaluates age, period, and cohort effects, revealing the complex interactions that shape disease trends over time (23). We use a pyramid chart to compare BMI-related PC mortality rates between men and women across countries.

## 3 Results

### 3.1 BMI-related PC mortality and DALY rates in the USA, Australia, and Germany, 2012 and 2021

According to **Table 1**, BMI-related PC mortality among adults aged 70 years or older increased from 2012 to 2021 in the United States, Australia, and Germany. The United States saw the largest rise; among those aged 95 years or older, BMI-related PC mortality increased from 3.79% to 4.66%. Australia also recorded substantial growth, with the 90–94 age group rising from 3.07% to 4.51%. Germany’s increase was smaller; among those aged 95 years or older, BMI-related PC mortality rose from 2.27% to 3.65%. Over the same period, DALYs among adults aged 70 years or older increased in all three countries. The largest gains occurred in the United States, where DALYs in the 70–74 age group rose from 60.28 to 66.09. Australia also showed notable increases in the 70–74 age group (34.90 to 43.33). In Germany, the increase was smaller but evident in those aged 95 years or older (18.72 to 30.11).

**Table 1:** BMI-related PC mortality and DALY rates in the USA, Australia, and Germany in 2012 and 2021.

Mortality	USA		Australia		Germany	
Age	2012	2021	2012	2021	2012	2021
70 to 74	2.99 (0.00 - 6.79)	3.28 (0.02- 7.26)	1.73 (-0.13- 4.35)	2.16 (-0.04 - 5.18)	1.65 (-0.36 - 4.81)	1.95 (-0.30- 5.73)
75 to 79	3.43 (-0.10 -8.15)	3.93 (-0.02 -8.94)	2.04 (-0.22 - 5.29)	2.59 (-0.12- 6.5)	1.76 (-0.57 - 5.39)	2.53 (-0.54 - 7.60)
80 to 84	2.65 (-0.51- 7.52)	3.4 (-0.24 - 8.95)	2.06 (-0.42 - 5.94)	2.68 (-0.35 - 7.41)	1.8 (-0.81 - 5.97)	2.10 (-0.72 - 6.73)
85 to 89	3.31 (-0.61- 9.33)	4.14 (-0.64- 10.27)	2.70 (-0.50 - 7.71)	3.57 (-0.42 - 9.55)	2.33 (-0.96 - 7.76)	2.34 (-0.72 - 7.34)
90 to 94	3.78 (-0.64 -10.27)	4.73 (-0.30- 12.20)	3.07 (-0.58 - 8.82)	4.51 (-0.47-12.15)	2.35 (-1.04 - 7.10)	3.35 (-1.03- 10.89)
95+	3.79 (-0.58 -10.35)	4.66 (-0.27 - 11.76)	3.15 (-0.60 - 8.98)	4.28 (-0.44-11.68)	2.27 (-0.89- 7.57)	3.65 (-1.22 - 11.69)
DALYs	USA		Australia		Germany	
Age	2012	2021	2012	2021	2012	2021
70 to 74	60.28 (-0.04 - 136.72)	66.09 (0.42 - 146.26)	34.90 (-2.53-87.42)	43.33 (-0.75-103.94)	33.04 (-7.29-96.53)	39.29 (-6.01-115.86)
75 to 79	55.24 (-1.55- 131.08)	63.38 (-0.35- 143.91)	32.78 (-3.59 - 85.54)	41.83 (-1.95-105.08)	28.37 (-9.16- 87.13)	40.38 (-8.54-121.79)
80 to 84	33.31 (-6.44 -94.58)	42.98 (-3.04 - 113.11)	25.87 (-5.26- 74.96)	33.82 (-4.38- 92.88)	22.49 (-10.17-74.78)	26.48 (-9.02-84.90)
85 to 89	33.12 (-6.05- 93.47)	41.45 (-2.72 - 107.98)	27.12 (-4.96 - 77.53)	35.84 (-4.18- 95.53)	23.36 (-9.66 - 77.66)	23.51 (-7.28 - 73.73)
90 to 94	33.11 (-5.59 - 89.93)	41.37 (-2.65 - 106.77)	26.90 (-5.06-77.14)	39.54 (-4.14-107.35)	20.61 (-9.11 - 62.24)	29.28 (-9.05 - 95.14)
95+	31.19 (-4.78 - 85.14)	38.38 (-2.26 - 96.88)	25.93 (-4.98 - 74.04)	35.06 (-3.60 -95.38)	18.72 (-7.31 - 62.38)	30.11 (-10.00-96.27)

### 3.2 ASR curves for the USA, Australia, and Germany over the past decade

**Figure 1:**
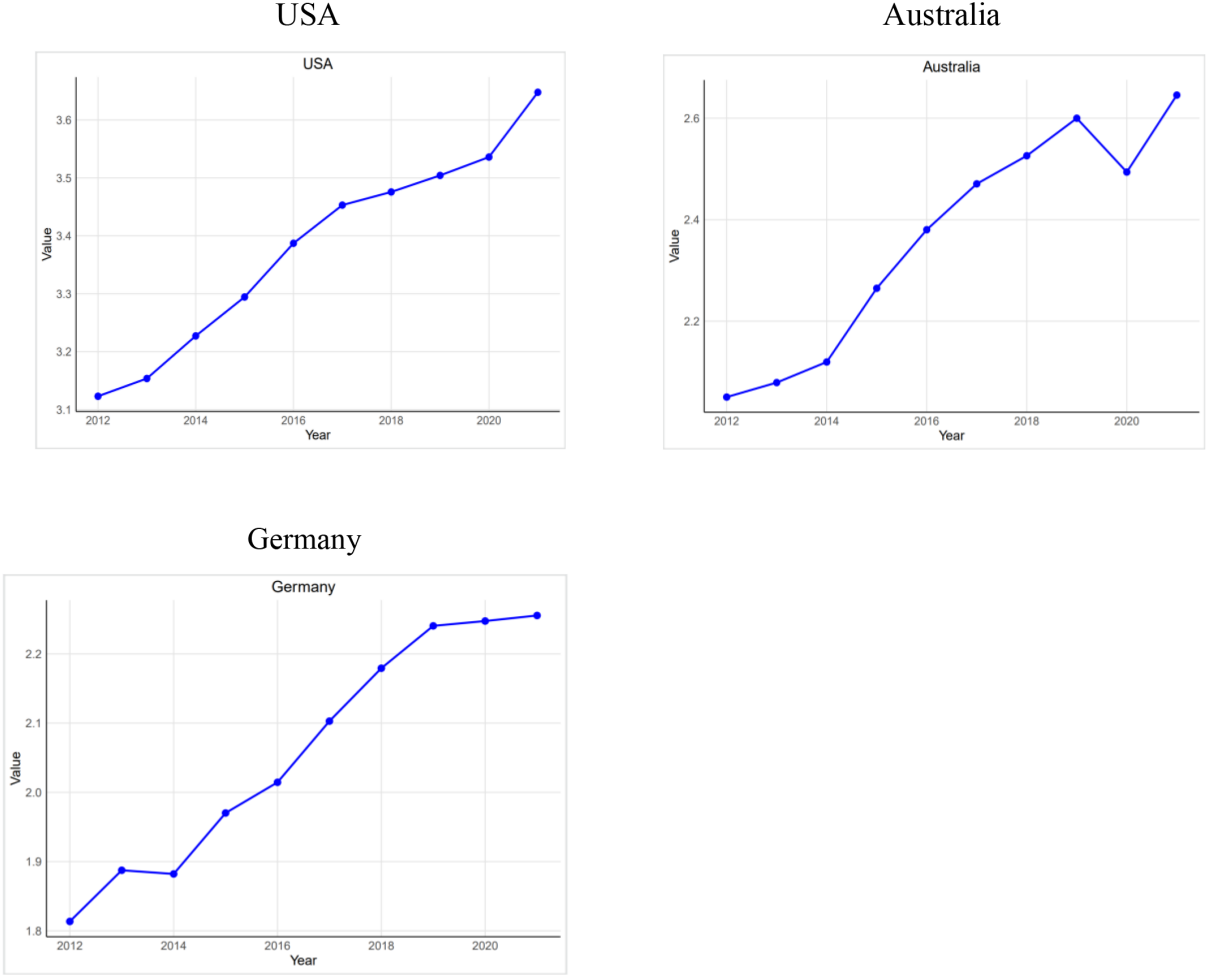
ASR rates of BMI-related PC mortality in the USA, Australia, and Germany from 2012 to 2021.

As shown in **Figure 1**, the USA has the steepest ASR increase, rising from approximately 3.12 in 2012 to 3.65 in 2021. Germany shows a flatter rise, from about 1.81 in 2012 to 2.26 in 2021. Australia falls between the two, increasing from about 2.05 in 2012 to 2.65 in 2021.

### 3.3 EAPC in the USA, Australia, and Germany over the past decade

In the USA, EAPCs for BMI-related PC mortality and DALYs were positive across all age groups, indicating upward trends. For ages 80–84 years, the EAPC for BMI-related PC mortality was 2.87 (95% CI 2.72–3.03) and for DALYs 2.93 (95% CI 2.78–3.08). In Australia, EAPCs were also positive across all age groups, with large increases; for ages 90–94 years, both BMI-related PC mortality and DALYs had an EAPC of 4.61 (95% CI 3.38–5.85). In Germany, EAPCs were positive in most age groups but near zero for ages 85–89 (0.76; 95% CI −0.28 to 1.81), indicating little change. Among those aged 95 years and older, EAPCs were markedly higher: 8.02 (95% CI 5.20–10.92) for BMI-related PC mortality and 8.06 (95% CI 5.18–11.03) for DALYs (**Table 2**).

**Table 2:** EAPC of BMI-Related PC Mortality and DALY rates in the USA, Australia, and Germany from 2012 to 2021.

	USA		Australia		Germany	
Age	Mortality	DALYs	Mortality	DALYs	Mortality	DALYs
70 to 74	0.96 (0.66-1.25)	0.96 (0.70-1.23)	2.54 (2.20-2.89)	2.53 (2.17-2.89)	1.65 (1.03-2.27)	1.78 (1.20-2.37)
75 to 79	1.47 (1.22-1.72)	1.50 (1.26-1.75)	3.06 (2.15-3.98)	3.09 (2.19-4.00)	4.70 (4.14-5.25)	4.54 (4.03-5.05)
80 to 84	2.87 (2.72-3.03)	2.93 (2.78-3.08)	3.01 (2.35-3.68)	3.07 (2.40-3.75)	1.95 (1.45-2.45)	2.03 (1.58-2.47)
85 to 89	2.61 (2.47-2.75)	2.62 (2.49-2.75)	3.36 (2.49-4.24)	3.34 (2.48-4.21)	0.76 (-0.28-1.81)	0.77 (-0.20-1.76)
90 to 94	2.42 (2.31-2.53)	2.42 (2.31-2.52)	4.61 (3.38-5.85)	4.61 (3.38-5.85)	4.04 (3.43-4.65)	4.03 (3.43-4.64)
95+	1.99 (1.64-2.34)	2.0 (1.65-2.35)	3.75 (2.46-5.05)	3.66 (2.36-4.98)	8.02 (5.20-10.92)	8.06 (5.18-11.03)

### 3.4 Joinpoint analysis of APC and AAPC across three countries

**Figure 2:**
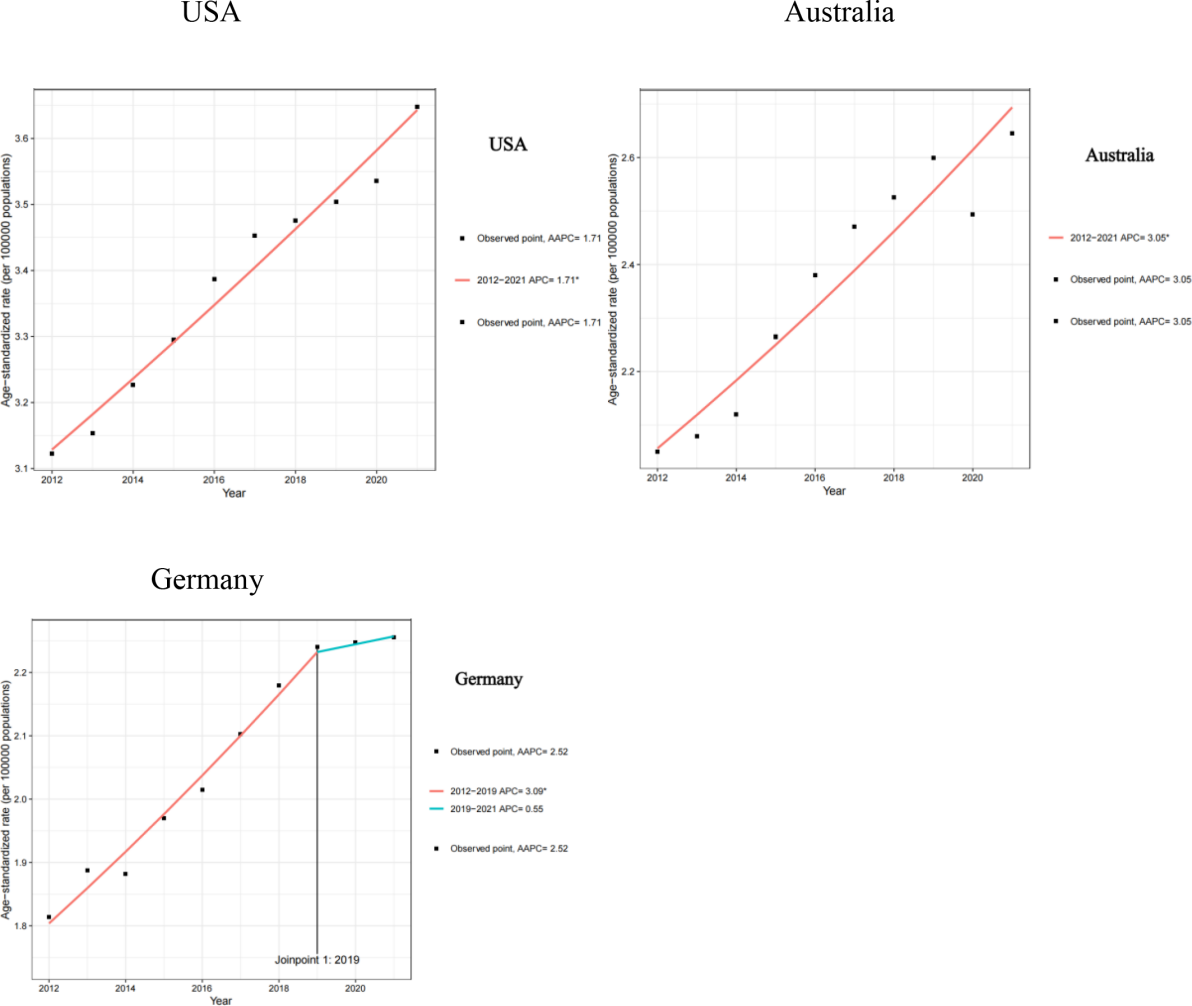
APC and AAPC of BMI-Related PC Mortality in the USA, Australia, and Germany from 2012 to 2021.

From 2012 to 2021, the USA had identical AAPC and APC values of 1.71. Australia showed higher trends, with both AAPC and APC at 3.05 over the same period. Germany displayed distinct temporal patterns: an APC of 3.09 during 2012–2019, slowing to 0.55 in 2019–2021, yielding an overall AAPC of 2.52. ASRs rose in all three countries, with the steepest increase in Australia. Germany’s growth slowed markedly after 2019, while the United States maintained a modest, steady rise (**Figure 2**).

### 3.5 Sex disparities in BMI-related PC mortality rates across three countries

**Figure 3:**
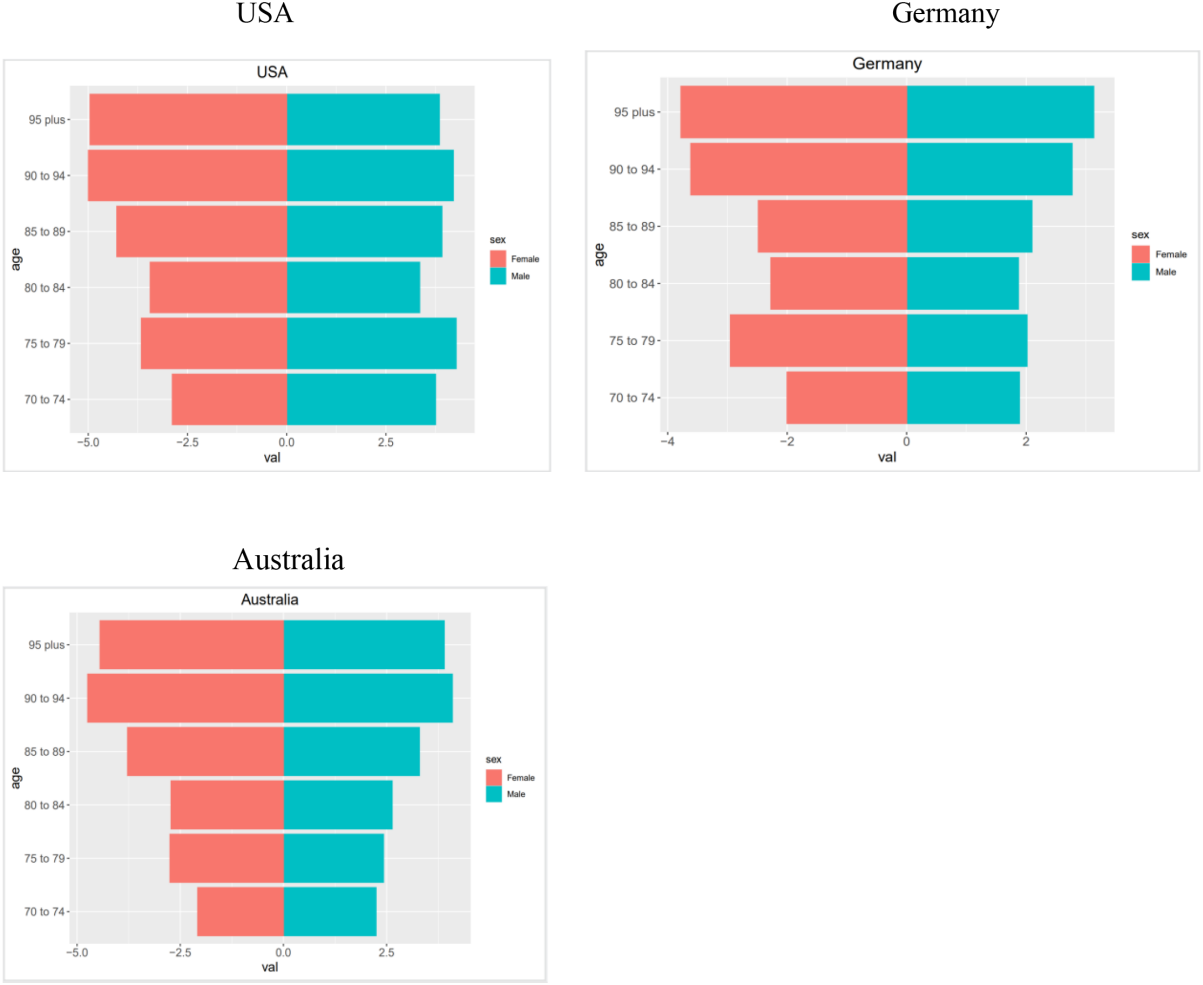
Pyramids of BMI-related PC mortality rates for the USA, Australia, and Germany in the year 2021.

In the USA, the BMI-related PC mortality rate for men aged 70–79 is generally higher than for women. Rates generally increase with age, peaking at 90–94 years; the rate is lowest at 80–84 years (3.40) and highest at 90–94 years (4.73). In Germany, BMI-related PC mortality rates are generally higher in women than in men; the rate is lowest at 70–74 years (1.95) and highest at 95 years and older (3.65). In Australia, men aged 70–74 have higher BMI-related PC mortality rates than women, whereas women have higher rates in other age groups. The rate is lowest at 70–74 years (2.16) and highest at 90–94 years (4.51) (**Figure 3**).

## 6 Discussion

Despite being relatively rare worldwide compared with other malignancies, PC is among the deadliest cancers (24). According to the National Cancer Center of China, the 5-year survival rate for PC is 7.2% (25). People with obesity tend to have elevated inflammatory markers. Consequently, they have a higher incidence of cancer (26). Gentiluomo et al. (27) used Mendelian randomization to show that BMI mediates the effect of sedentary behavior on PC risk. Therefore, BMI is an important risk factor for PC. We analyzed the epidemiologic characteristics of BMI-related PC among adults aged 70 years or older in the United States, Germany, and Australia from 2012 to 2021. Overall, BMI-related PC mortality and DALYs increased in all three countries, reflecting the health challenges of aging populations.

Next, we calculated the ASR and plotted the ASR curves for different populations. The ASR in the USA grew the fastest, indicating that the health burden of its elderly population grew the fastest and that more active public health policies were needed. Germany’s ASR grew more slowly, reflecting the effectiveness of its medical system and social policies, but continued attention should be paid to the health of its elderly population. Australia’s ASR growth was intermediate, suggesting the need to strengthen health management and resource allocation to meet the challenges of ageing. Overall, the rising trend in ASR in the three countries reflects the increasing health burden of the elderly population, but the rate of growth and the reasons for it are different. Countries should formulate appropriate health policies and interventions according to their own conditions to meet the health challenges of an aging society.

Next, we calculated the age-standardized rate (ASR) and plotted trends by country. The United States had the fastest increase, indicating the most rapid growth in the health burden among older adults and underscoring the need for more proactive public health policies. Germany’s ASR rose more slowly, suggesting effective health and social systems, though continued attention to older adults’ health remains necessary. Australia’s ASR increase was intermediate, indicating a need to strengthen health management and resource allocation to address population aging. Overall, rising ASRs across the three countries show a growing health burden among older adults, although rates and underlying drivers vary. Countries should tailor policies and interventions to their specific contexts to meet the challenges of an aging society.

We then used EAPC to compare rates of change in burden across age groups. In the United States, the burden among older adults rose each year, especially among those aged 80 years and older, likely reflecting higher chronic disease prevalence, pressure on health services, and rapid population aging. In Australia, the burden also increased, particularly in the older age groups, reflecting accelerated aging and challenges in chronic disease management. In Germany, the burden increased markedly among those aged 95 years and older but remained relatively stable in other groups, suggesting benefits of its robust health and social systems; nevertheless, the oldest age groups require continued attention. Countries should strengthen health management for older adults and tailor resource allocation to local conditions to address the growing burden of aging.

We used joinpoint regression to estimate the annual percent change (APC) and average annual percent change (AAPC). In Australia, the ASR rose steadily (AAPC 3.05%), suggesting annual increases in BMI-related PC mortality, likely driven by rising obesity and accelerated population aging. In Germany, the ASR increased rapidly from 2012 to 2019 (APC 3.09%) and then slowed markedly from 2019 to 2021 (APC 0.55%), possibly reflecting strengthened obesity prevention and management and advances in medical technology. In the USA, the ASR also trended upward (AAPC 1.71%), indicating ongoing increases in BMI-related PC mortality, likely linked to high obesity prevalence and an aging population. Countries should strengthen obesity prevention and management and promote healthy lifestyles to reduce BMI-related PC mortality. Australia and the United States may require more proactive public health interventions, while Germany can continue refining existing strategies to sustain slower growth.

Lastly, we used a population pyramid to compare disease distribution by sex across age groups. Many studies (8, 28) have found that higher BMI is strongly associated with PC in men. In the United States, recent increases in PC incidence and mortality are driven mainly by pancreatic ductal adenocarcinoma (29). Pancreatic adenocarcinoma is more common among men than among women across several regions (30). In the USA, BMI-related PC mortality is higher among men and older adults, likely reflecting high obesity prevalence and an aging population. In Australia, the pattern is similar, with a heavier burden among men and older adults, possibly due to higher male obesity and chronic disease rates. In Germany, the burden is higher among women, potentially linked to higher obesity prevalence among women (31). Finally, we examined potential mechanisms linking high BMI to PC progression. Li et al. (32) found that a higher preoperative neutrophil-to-lymphocyte ratio was associated with poorer prognosis in pancreatic cancer. Van Bruggen et al. (33) found that neutrophil peptidylarginine deiminase 4 may exacerbate obesity-induced chronic inflammation. Yang et al. (34) found that fatty acid binding protein 4 in macrophages promotes obesity-related pancreatic cancer progression by affecting the NOD-, LRR- and pyrin domain-containing (NLRP)3/interleukin (IL)-1β axis. Future research should investigate the links among BMI, neutrophils, and pancreatic cancer. Pita-Grisanti et al. (35) found that physical activity reduces inflammation and delays the development of obesity-related pancreatic ductal adenocarcinoma. Thus, regular exercise may lower pancreatic cancer risk. Divergent trends in pancreatic cancer BMI-related PC mortality and DALYs in the U.S., Australia, and Germany stem from multiple factors (36, 37). A key factor is the varying prevalence of obesity (12). As a leading risk factor, its global rise correlates with increasing age-standardized BMI-related PC mortality and DALY rates. These rates are often higher in nations with greater healthcare access, potentially reflecting their obesity demographics. The burden is further exacerbated by healthcare disparities. High Socio-demographic Index (High-SDI) regions report a greater risk burden, yet racial and socioeconomic barriers limit access to quality care, impacting survival and resource allocation (38, 39). Additionally, differences in national lifestyle factors contribute to the heterogeneity. These interconnected factors underscore the need for tailored national interventions to improve data collection and public health policies (40).

This study presents the first analysis of BMI-related pancreatic cancer among older adults and the first comparison of epidemiologic characteristics across the United States, Australia, and Germany. These findings provide a foundation for future policy development and implementation.

However, this study also has many shortcomings because the GBD database only has BMI indicators and no other more accurate indicators of obesity. Maina’s et al. (41) study found that indicators of abdominal obesity are a more important risk factor for pancreatic cancer compared to overall obesity. Jin et al. (42) study found that the skeletal muscle mass/BMI ratio can better reflect the 3–5-year disease-free survival rate. Therefore, it can be combined with other databases for further research in the future.

The current analysis is also limited by the Global Burden of Disease (GBD) data’s lack of systematic adjustment for healthcare system characteristics—such as resource accessibility, insurance disparities, and treatment strategies. Disparities in healthcare workforce distribution and shortages have been shown to constrain PC diagnosis and treatment capacity, potentially affecting the accuracy of BMI-related PC mortality and DALY estimates (43). In the elderly population, cross-national differences in healthcare resources may explain variations in trends. However, the GBD does not independently quantify these factors, introducing a risk of omitted variable bias.

## 7 Conclusion

This study compared the burden of PC attributable to high BMI among adults aged 70 years or older from 2012–2021 in the United States, Australia, and Germany. The United States showed the steepest increases in BMI-related PC mortality and DALYs, followed by Australia, while Germany experienced milder growth. Australia’s ASR rose fastest (AAPC = 3.05%); Germany’s growth slowed after 2019 (0.55%); and the United States increased steadily (1.71%). These findings underscore the need for country-specific obesity interventions tailored to aging populations.

## Data Availability

The original contributions presented in the study are included in the article/supplementary material. Further inquiries can be directed to the corresponding author.
